# Perforated Meckel’s Diverticulum as a Rare Cause of Pneumoperitoneum in a Post-pubescent Adolescent: A Case Report

**DOI:** 10.7759/cureus.70510

**Published:** 2024-09-30

**Authors:** Fiona Beltran, Cornelia Griggs, Alyssa Stetson

**Affiliations:** 1 Medical School, Tufts University School of Medicine, Boston, USA; 2 Pediatric Surgery, Massachusetts General Hospital, Boston, USA

**Keywords:** acute surgical abdomen, laparoscopic resection, meckel’s diverticulum, perforation, pneumoperitoneum

## Abstract

Meckel’s diverticulum (MD) is known to cause surgical emergencies including intussusception, obstruction, and bleeding, but rarely results in perforation with pneumoperitoneum. Symptomatic MD is rare but most commonly presents in early childhood. We report a case of a 17-year-old male who presented with peritonitis and radiographic imaging demonstrating pneumoperitoneum and inflammation near the appendix and terminal ileum. Intraoperative findings revealed a perforated MD. A perforated MD may mimic a diagnosis of complicated appendicitis in the adolescent and adult population and should be considered in the differential diagnosis of patients presenting with pneumoperitoneum and an acute surgical abdomen.

## Introduction

Meckel’s diverticulum (MD) results from failed obliteration of the omphalomesenteric duct (OMD) and is the most common congenital anomaly of the gastrointestinal tract [1]. Histopathologically, MD is a true diverticulum containing all three layers of the small bowel wall [2]. The diverticulum itself may be lined by ileal mucosa, heterotopic gastric or pancreatic tissue [3]. Ectopic acid secretion, as in the gastric mucosa subtype, may lead to ulceration of intestinal mucosa and bleeding [4]. While usually asymptomatic, complications of MD can be severe. Among pediatric patients, the prevalence and type of severe complications include bowel obstruction (40%), intussusception (14%), gastrointestinal bleeding (31%), and inflammation (12%) [5]. However, spontaneous MD perforation is rare and can mimic acute appendicitis.

## Case presentation

**Figure 1:**
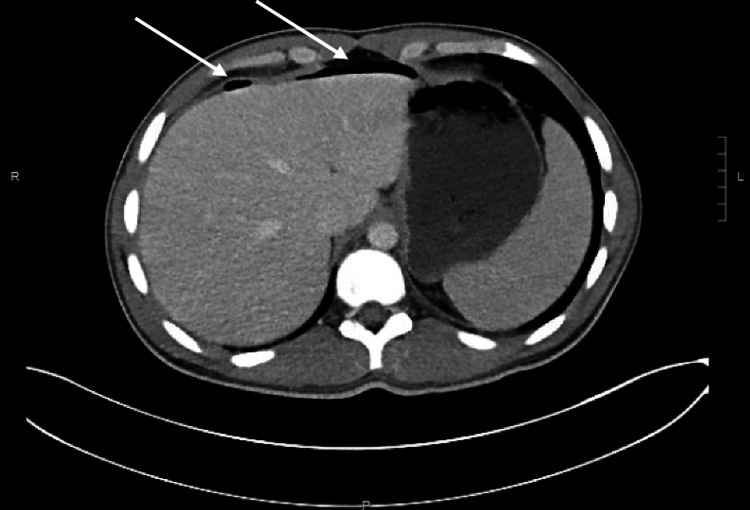
Pneumoperitoneum on CT imaging Arrows indicating pneumoperitoneum. CT: Computed tomography

**Figure 2:**
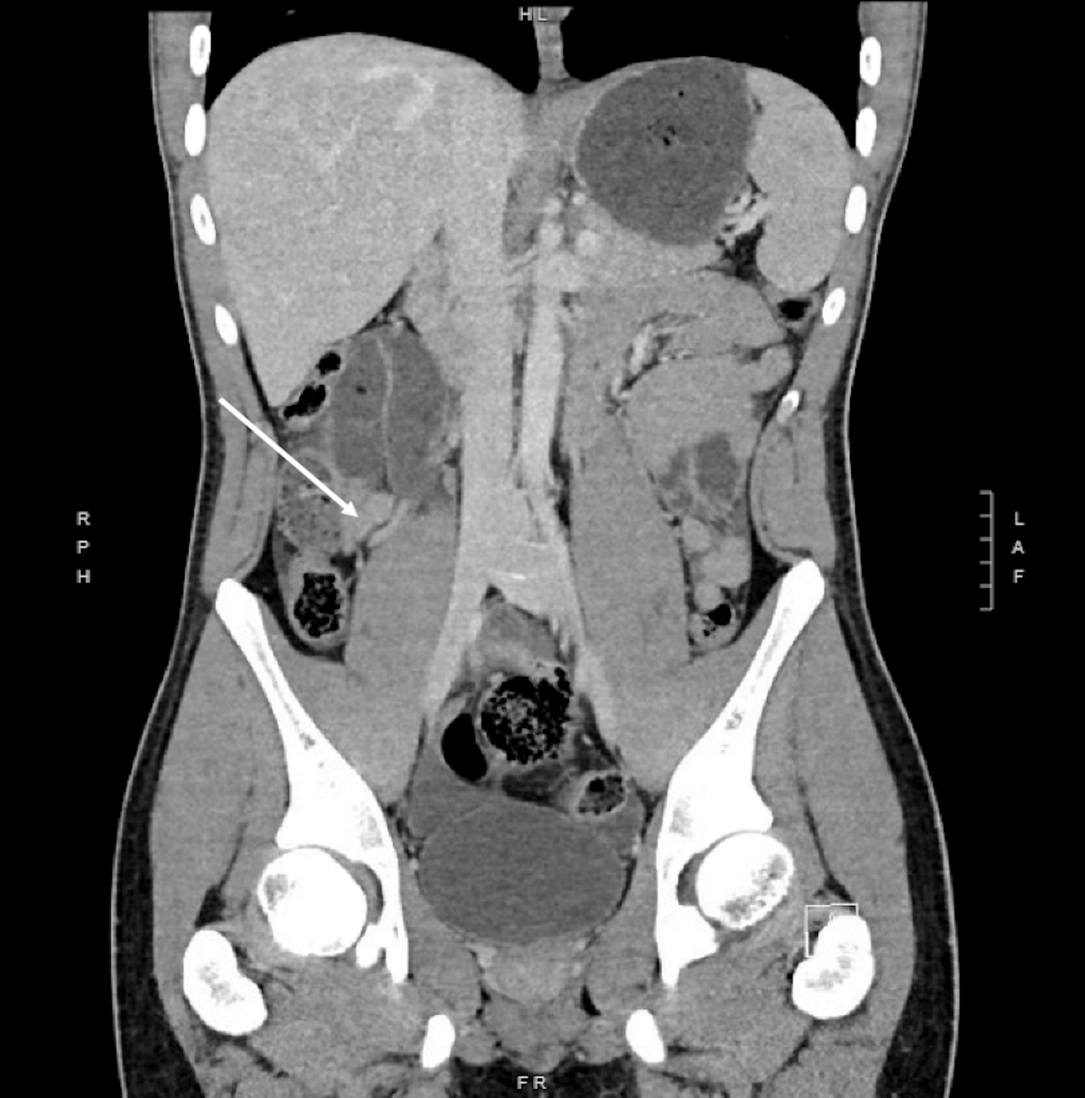
Noninflamed appendix on CT imaging Coronal CT image with arrows indicating noninflamed appendix. CT: Computed tomography

A 17-year-old healthy adolescent male with no significant past medical history presented to the emergency department with severe abdominal pain and tachycardia. He had no smoking history and no recent nonsteroidal anti-inflammatory drug (NSAID) use. He reported three days of periumbilical pain that was localized to the right lower quadrant. He denied nausea, vomiting, diarrhea, constipation, hematuria, and dysuria. In the emergency room, he was afebrile, hypertensive, and without leukocytosis. Physical exam findings revealed abdomen diffusely tender to palpation with guarding in all four abdominal quadrants. An abdominal ultrasound revealed a complex fluid collection in the right lower quadrant and pelvis that was of unclear etiology. Computed tomography (CT) was recommended and demonstrated pneumoperitoneum (Figure 1) and both small and large bowel wall thickening including the terminal ileum. Radiological imaging further demonstrated a noninflamed appendix (Figure 2). 

Preoperative intravenous (IV) piperacillin and tazobactam were started and the patient was taken to the operating room for diagnostic laparoscopy. Intraoperative findings revealed a normal appendix with no inflammation. Pus was seen freely throughout the peritoneum and was concentrated in the right and left lower quadrants and the pelvis. The bowel was run, revealing a 1.5 cm perforation at the base of an MD located approximately 50 cm proximal to the ileocecal valve. Given the wide area of perforation at its base, it was determined that diverticulectomy alone would result in significant narrowing of the ileal lumen. As such, the decision was made to convert to open via a lower midline incision and to perform a small bowel resection (8 cm of bowel was removed) with a stapled side-to-side anastomosis to eliminate the area of perforation and necrosis. One Jackson-Pratt (JP) drain was placed in the left lower quadrant.

The patient tolerated the procedure well and the nasogastric tube was removed on the first postoperative day. On postoperative day two, the JP drain was removed, and he transitioned from IV antibiotics to oral antibiotics. His diet was advanced after evidence of the return of bowel function.

Final pathology confirmed heterotopic gastric mucosa oxyntic type consistent with an MD.

## Discussion

We report a case of a previously healthy 17-year-old who presented with generalized peritonitis secondary to perforated MD. The preoperative diagnosis was perforated appendicitis based on radiographic evidence of pneumoperitoneum and inflammation near the appendix and terminal ileum. Intraoperative findings revealed perforated MD, which was managed with small bowel resection and stapled side-to-side anastomosis.

MD is the most common congenital malformation of the gastrointestinal tract and occurs due to the persistence of the OMD in the embryonic stage [1]. MD is largely asymptomatic and risk factors for symptomatic diverticulum include age younger than 50 years, male sex, diverticulum greater than 2 cm in length, and the presence of ectopic tissue [5]. Symptomatic MD typically presents in children under two years and incidence declines with age [5]. The most common complications of MD include intestinal bleeding, obstruction, and intussusception [6]. Perforation is rare and reported in 10% of MD complications [7]. Heterotopic mucosa has been reported in 59% of symptomatic resected diverticulum [7], however, its presence is not a prerequisite for perforation [8]. Symptomatic MD, as in this case, should be treated by surgical resection. Farah et al. report the management of spontaneous MD perforation with diverticulectomy and ileostomy [9]. Huang et al. summarized that 73% of cases of diverticulitis and/or perforated diverticulum were managed open, while 24% were managed laparoscopically [10]. These numbers reflect national trends in the surgical management of all symptomatic MDs regardless of primary or secondary diagnoses [11].

Perforated MD is rare, and diagnosis is challenging given its general symptomatic presentation and nonspecific imaging. Perforated MD may mimic complicated appendicitis and should be considered in the evaluation of an otherwise healthy young adult with an acute surgical abdomen. Surgical management of true appendicitis should include a full inspection of the bowel to evaluate for MD. Because many adult treatment centers are considering nonoperative management of appendicitis, a degree of suspicion should be maintained for alternate diagnoses, especially in the setting of pneumoperitoneum. 

There is limited data on the surgical management of perforated MDs. This case demonstrates a successful example of laparoscopic converted to open management of perforated MD through small bowel resection and stapled side-to-side anastomosis. Preoperative consideration of perforated MD in cases of acute surgical abdomen may guide surgical management and preoperative planning of laparoscopic versus open approach.

## Conclusions

MD complications are rare and challenging to diagnose preoperatively. This case demonstrates that perforated MD may clinically mimic complicated appendicitis in a previously healthy young adult. Although rare, MD complications should be considered in the evaluation of acute surgical abdomen accompanied by pneumoperitoneum. Preoperative consideration of MD may guide surgical decision-making and overall treatment approach.
